# Supplementary figures and images for: Activity-mediated accumulation of potassium induces a switch in firing pattern and neuronal excitability type
Source: PLoS Comput Biol. 2021 May 27;17(5):e1008510. doi: 10.1371/journal.pcbi.1008510 (PMC8205125; doi:10.1371/journal.pcbi.1008510)

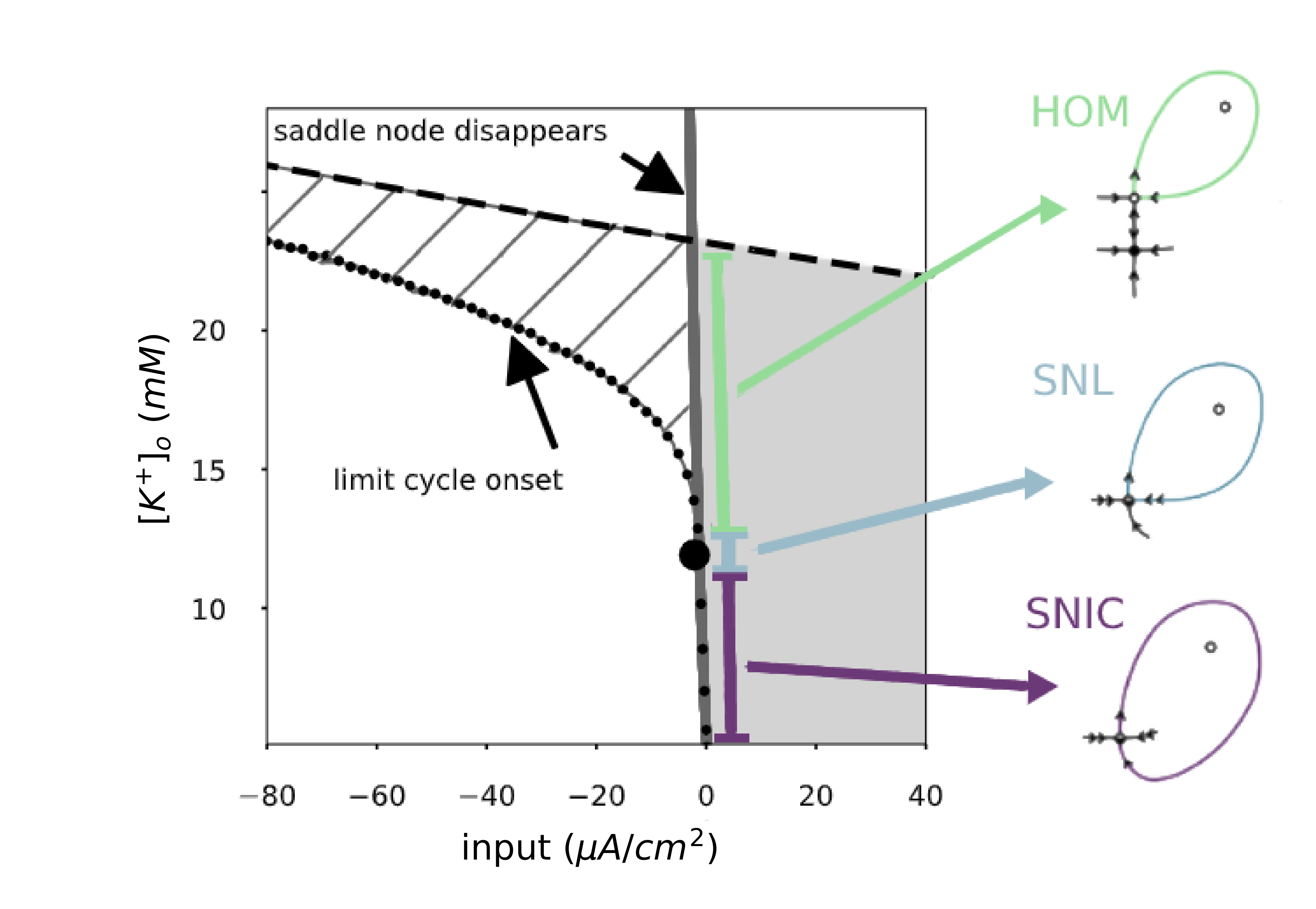

Supplement: S1 Fig — From bottom to top; SNIC (saddle-node on invariant circle): Purple, SNL (Saddle-node-loop): Blue; HOM (saddle homoclinic orbit): Green. In the SNIC regime the stable node collides with an unstable node, giving rise to a saddle node. The limit cycle orbit passes through the saddle node, the trajectory leaves the saddle node along the semi-stable manifold. After one period trajectory approaches the saddle node along the same semi-stable manifold. At the SNL point, trajectories leave the saddle node along the semi-stable manifold as in the SNIC case, but after one period those trajectories approach the saddle node along the strongly stable manifold. Notice that the SNL orbit is smaller than the SNIC orbit, and has a shorter period. In the HOM regime a stable node and a limit cycle coexist. External perturbations shift the state of the system from the stable node to the attraction domain of the limit cycle attractor. (TIFF) [file pcbi.1008510.s001.tiff]
