# Supplementary material for: Activity-mediated accumulation of potassium induces a switch in firing pattern and neuronal excitability type
Source: PLoS Comput Biol. 2021 May 27;17(5):e1008510. doi: 10.1371/journal.pcbi.1008510 (PMC8205125; doi:10.1371/journal.pcbi.1008510)
Supplement: S1 Text — Fig A. Changes in the conductance of the delayed rectifier potassium current (gK) distorts the bistable region portrayed in Fig 3A. Same bifurcation diagram portrayed in Fig 3A for different gK. Here the curves correspond to the delayed rectifier conductance of gK; 100, 200, 300, and 340 msiemens/cm2. As gK increases, the limit cycle onset, and the depolarization block lines are shifted towards higher extracellular potassium concentrations. Fig B. Changes in the leak conductance (gL) distorts the bistable region portrayed in Fig 3A. Same bifurcation diagram portrayed in Fig 3A for different gL. Here the curves correspond to leak conductances of gL; 0.01, 0.1, 0.5, and 1.0 msiemens/cm2. As gL increases, the bistable region is shifted towards higher extracellular potassium concentrations. Another effect of more leaky neurons, is that the dependence of the spiking threshold on extracellular potassium is more prominent. Fig C. Extracellular potassium and [K+]o pump’s sensitivity (Ksens) dependent bistable area. Same bifurcation diagram portrayed in Fig 6 for different [K+]o pump’s sensitivity. Here 0,0.1,0.2 and 0.5 1/mM sensitivities to [K+]o (Ks) are portrayed and [K+]s is fixed to 4mM for all curves, the expression of the pump that was used here resembles isoform α2 (eq A in S1 Text). Ks distorts the saddle node bifurcation line, curving it towards more depolarized currents, i.e., shifting the spiking threshold towards higher input currents. (PDF) [file pcbi.1008510.s002.pdf]

Activity-mediated accumulation of potassium induces a switch in firing pattern and neuronal excitability type

Susana Andrea Contreras[1,2], Jan-Hendrik Schleimer[1,2], Allan T. Gulledge[3], Susanne Schreiber*[1,2]

1 Institute for Theoretical Biology, Humboldt-University of Berlin, Berlin, Germany.

2 Bernstein Center for Computational Neuroscience Berlin, Berlin, Germany.

3 Molecular and Systems Biology, Geisel School of Medicine at Dartmouth College, Hanover, New Hampshire, United States.

*Corresponding Author Susanne Schreiber

E-mail: s.schreiber@hu-berlin.de

Supporting information

S1 Text: Robustness of results to model choice

Parameter choice

As mentioned in the main text, there are parameters that can shift the exact extracellular potassium concentration that induces the bistability. The effects of changing the maximum conductance of potassium and the leak conductance on the bistable region are show in Figs A and B.

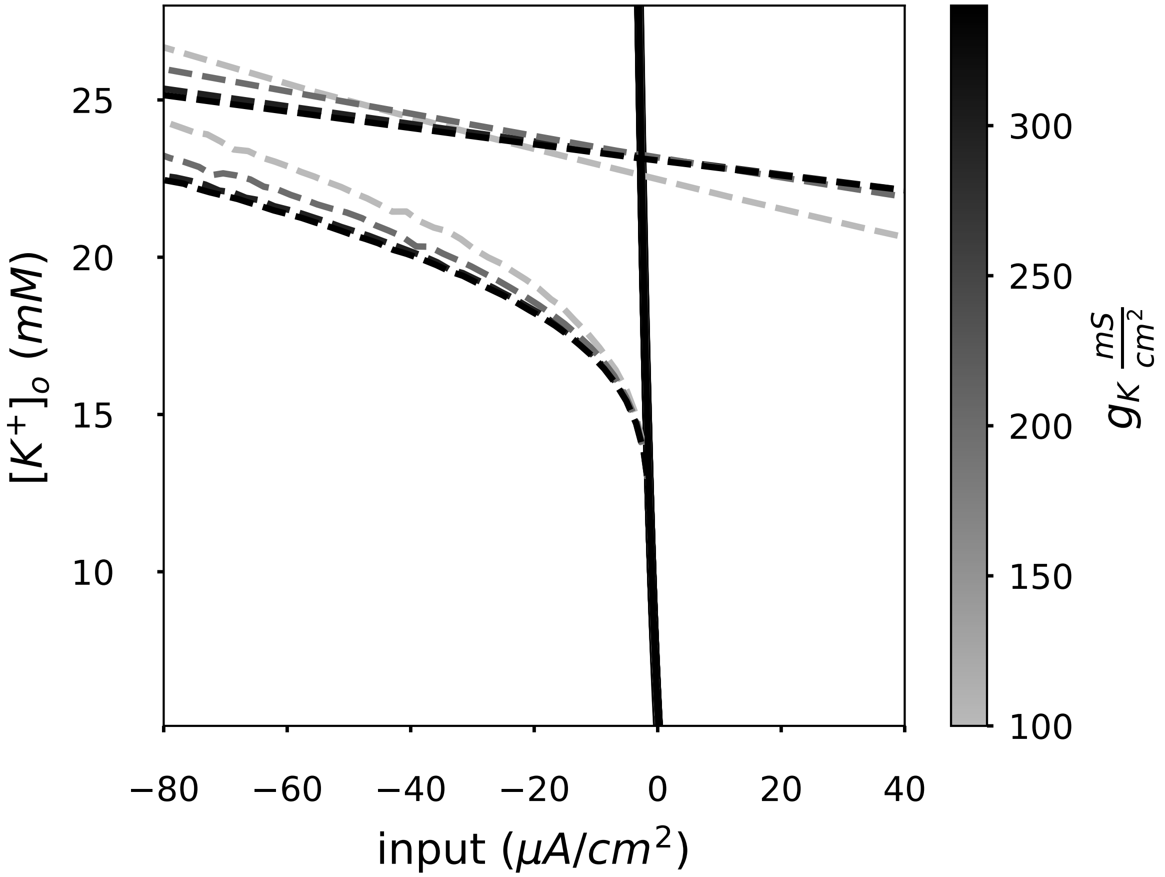

Fig A. Changes in the conductance of the delayed rectifier potassium current (g_K) distorts the bistable region portrayed in Fig.3A. Same bifurcation diagram portrayed in Fig.3A for different g_K. Here the curves correspond to the delayed rectifier conductance of g_K; 100, 200, 300, and 340 $msiemens/cm^2$. As g_K increases, the limit cycle onset, and the depolarization block lines are shifted towards higher extracellular potassium concentrations.

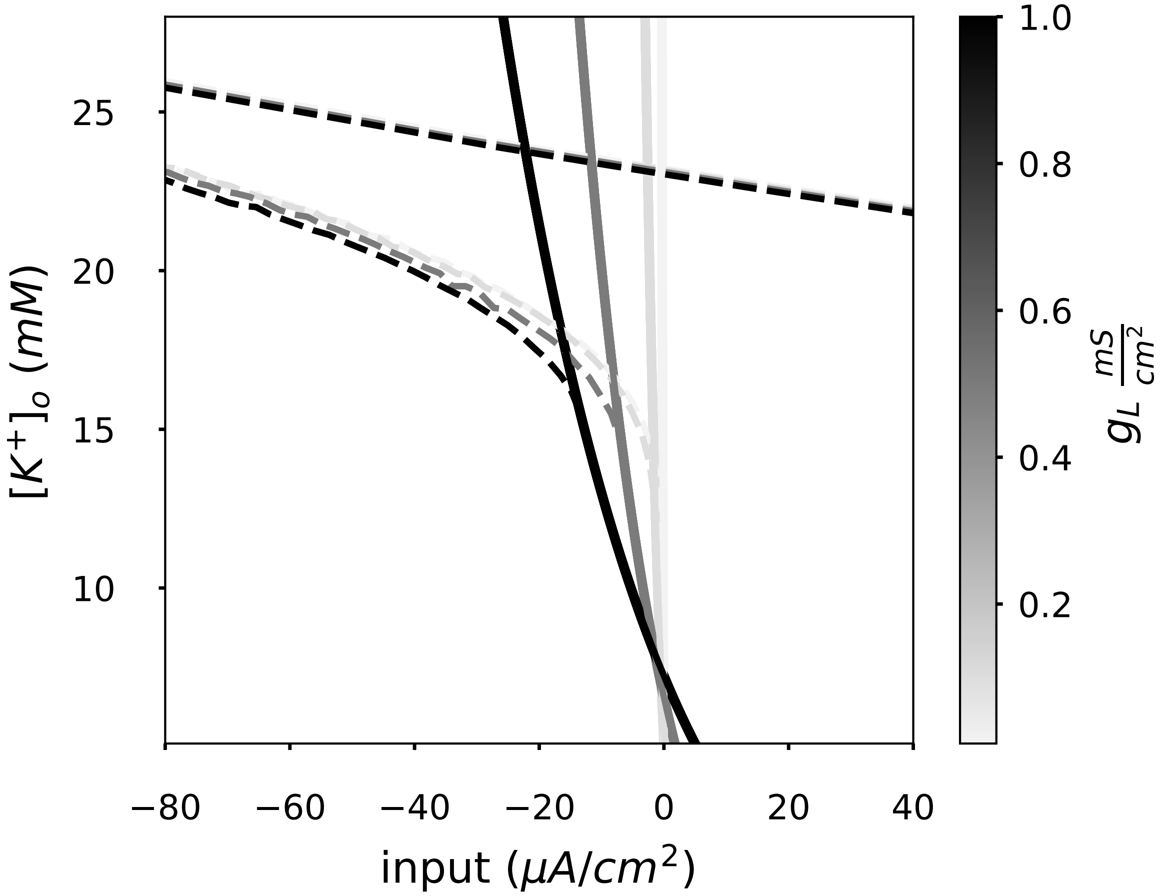

Fig B. Changes in the leak conductance (g_L) distorts the bistable region portrayed in Fig.3A. Same bifurcation diagram portrayed in Fig.3A for different g_L. Here the curves correspond to leak conductances of g_L; 0.01, 0.1, 0.5, and 1.0 $msiemens/cm^2$. As g_L increases, the bistable region is shifted towards higher extracellular potassium concentrations. Another effect of more leaky neurons, is that the dependence of the spiking threshold on extracellular potassium is more prominent.

Pump choice

The pump choice in the model represents an analog of the $\alpha 3$ isoform of the Na-K-ATPase, which reacts very strongly to intracellular sodium changes, but is rather insensitive to potassium in the range we study ($[\mathrm{K}^+]_o$ 4-20 mM) [1].

$$I_{\mathrm{pump}} = \begin{cases} 0 & [\mathrm{Na}^+]_i \leq [\mathrm{Na}]_s \\ \frac{I_{\mathrm{maxp}}}{1+\exp(k_{\mathrm{Na}}([\mathrm{Na}^+]_i - [\mathrm{Na}]_s))} \frac{2}{1+\exp(-K_s([\mathrm{K}^+]_o - [\mathrm{K}^+]_s))} & [\mathrm{Na}^+]_i > [\mathrm{Na}]_s \end{cases} \tag{A}$$

To understand how our pump choice determines the qualitative results presented, we performed a bifurcation analysis to the model described in the main text replacing the pump expression to one that resembles pump isoform $\alpha 2$. The main difference between the pump expression in the main text and the one used to resemble $\alpha 2$ (eq. A), is the pump sensitivity to extracellular potassium. Fig C illustrates how the pump sensitivity to $[K^+]_o$ shifts the spiking threshold towards higher input currents as $[K^+]_o$ accumulates.

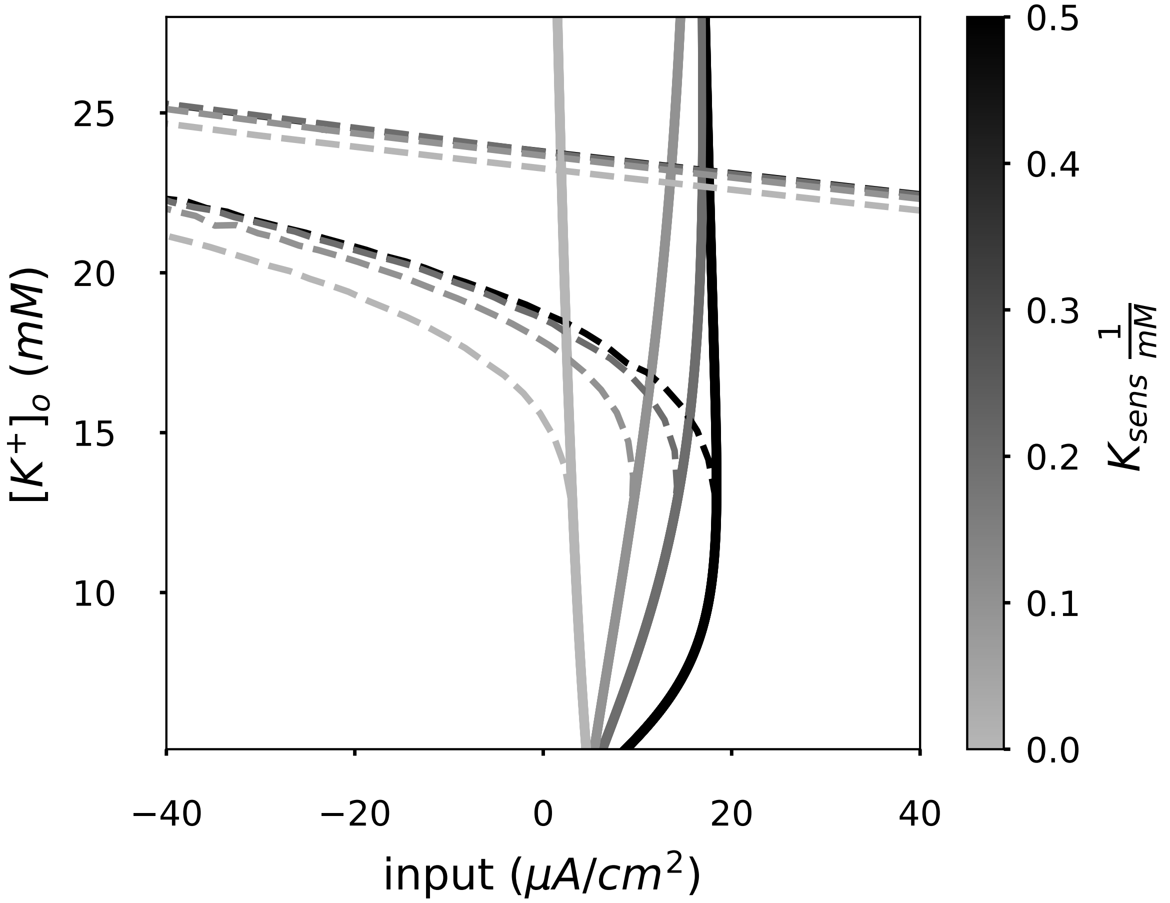

Fig C. Extracellular potassium and $[K^+]_o$ pump's sensitivity (K_{sens}) dependent bistable area. Same bifurcation diagram portrayed in Fig. 6 for different $[K^+]_o$ pump's sensitivity. Here 0,0.1,0.2 and 0.5 $1/mM$ sensitivities to $[K^+]_o$ (K_{s}) are portrayed and $[\text{K}^+]_s$ is fixed to 4 mM for all curves, the expression of the pump that was used here resembles isoform α_2 (eq. A). K_{s} distorts the saddle node bifurcation line, curving it towards more depolarized currents, i.e shifting the spiking threshold towards higher input currents.

References

1. Crambert G, Hasler U, Beggah AT, Yu C, Modyanov NN, Horisberger JD, et al. Transport and Pharmacological Properties of Nine Different Human Na,K-ATPase Isozymes*; 2000. Available from: http://www.jbc.org/.
