# Supplementary material for: Activity-mediated accumulation of potassium induces a switch in firing pattern and neuronal excitability type
Source: PLoS Comput Biol. 2021 May 27;17(5):e1008510. doi: 10.1371/journal.pcbi.1008510 (PMC8205125; doi:10.1371/journal.pcbi.1008510)
Supplement: S2 Text — Fig D. Slow decay of spike amplitudes. Voltage recording of a neuron experiencing depolarizing pulses applied at 40 Hz. The fast and slow time constants of amplitude decay were τfast = 410(ms) and τslow = 13.6(sec), respectively. Notice that the peak of the last spike fails to recover to the initial amplitude after the one-second-long hyper-polarizing pulse. Fig E. Distribution of time scales of the double exponential decay (Eq 13) of the spike amplitude. Two protocols were used to measure the time scales of spike amplitude decay, an example of the “40 Hz Depolarizations” is shown in Fig D in S2 Text, and an example of the “Hyperpolarization” is shown in Fig 5. Notice that the distribution of τslow is independent of the protocol used. Table A. Summary of the distribution of the best fit of the parameters for each of the 50 traces. Depolarizing pulses applied at a 40Hz rate. Table B. Summary of the distribution of the best fit of the parameters for each of the 73 traces. Hyperpolarizing pulses. (PDF) [file pcbi.1008510.s003.pdf]

Activity-mediated accumulation of potassium induces a switch in firing pattern and neuronal excitability type

Susana Andrea Contreras[1,2], Jan-Hendrik Schleimer[1,2], Allan T. Gulledge[3], Susanne Schreiber*[1,2]

1 Institute for Theoretical Biology, Humboldt-University of Berlin, Berlin, Germany.

2 Bernstein Center for Computational Neuroscience Berlin, Berlin, Germany.

3 Molecular and Systems Biology, Geisel School of Medicine at Dartmouth College, Hanover, New Hampshire, United States.

*Corresponding Author Susanne Schreiber
E-mail: s.schreiber@hu-berlin.de

Supporting information

S2 Text: Timescale of spike amplitude decay

As mentioned in the main text, extended activation of rodent cortical neurons led to a slow spike amplitude reduction (Fig 5 in the main text, and Fig D).

Stimulation protocol

Rodent cortical neurons were activated for 40 seconds using short (2 ms) depolarizing current pulses (3 nA) generated at 40 Hz (refer to Fig D).

Finding best fit of spike amplitude decay

These neurons exhibited a slow and progressive reduction in spike amplitude that was best fit by a double exponential decay. The fast and the slow components of the spike amplitude decay were calculated by fitting the time dependent spike-voltage-peak to a double exponential function,

$$D_{fast} \exp[\frac{-t}{\tau_{fast}}] + D_{slow} \exp[\frac{-t}{\tau_{slow}}] + D_{ss}.(\mathrm{B})$$

D_{ss} represents the spike peak at the steady state (when the spike peak is not reducing anymore). The sum $D_{ss} + D_{fast} + D_{slow}$ yields the spike peak right at stimulus onset. τ_{fast} is the fast time

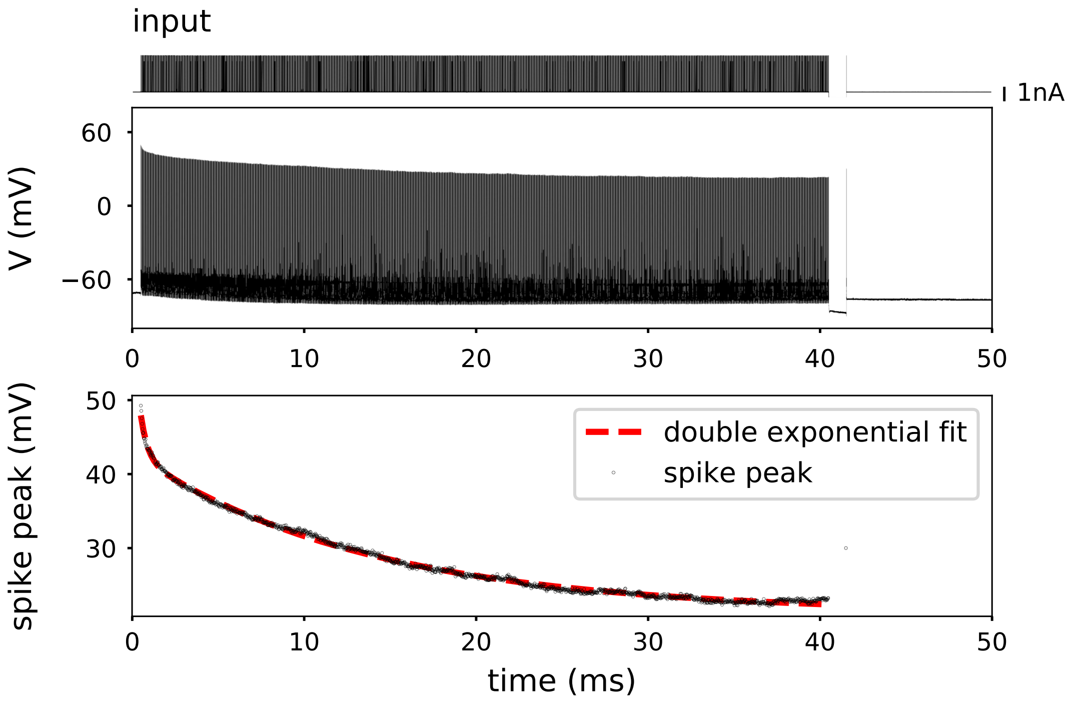

Fig D. Slow decay of spike amplitudes. Voltage recording of a neuron experiencing depolarizing pulses applied at 40 Hz. The fast and slow time constants of amplitude decay were $\tau_{fast} = 410(ms)$ and $\tau_{slow} = 13.6(sec)$, respectively.. Notice that the peak of the last spike fails to recover to the initial amplitude after the one-second-long hyper-polarizing pulse.

	τ_{fast} $[ms]$	τ_{slow} $[ms]$	D_{fast} $[mV]$	D_{slow} $[mV]$	D_{ss} $[mV]$
count	48.000000	48.000000	48.000000	48.000000	4.800000e+01
mean	480.314157	17728.034470	5.906181	20.379248	2.352945e+01
std	676.504019	7919.924120	2.470383	6.366880	1.147889e+01
min	61.687083	4312.710113	0.000001	7.860144	6.323943e-07
25%	170.353968	12050.396908	4.334064	17.144954	1.672852e+01
50%	232.372743	16797.597216	5.823583	20.102315	2.359666e+01
75%	368.297499	21156.877421	7.097274	22.357364	3.093022e+01
max	3083.939293	45815.441228	14.308320	48.164289	5.970884e+01

Table A. Summary of the distribution of the best fit of the parameters for each of the 48 traces. Depolarizing pulses applied at a 40Hz rate. See

	τ_{fast} $[ms]$	τ_{slow} $[ms]$	D_{fast} $[mV]$	D_{slow} $[mV]$	D_{ss} $[mV]$
count	73.000000	73.000000	73.000000	73.000000	73.000000
mean	935.652651	15707.653647	5.064957	7.737310	40.589393
std	599.644611	9936.369466	2.492782	4.081336	7.589756
min	125.198826	1485.350564	0.965347	0.000004	0.000024
25%	466.154928	10033.043848	3.045424	5.644995	36.885021
50%	745.856489	13876.630769	4.882892	7.078889	42.152299
75%	1319.313755	18294.743647	6.750978	8.840077	45.033475
max	2436.623039	52548.189654	11.642939	32.905853	52.764149

Table B. Summary of the distribution of the best fit of the parameters for each of the 73 traces. Hyperpolarizing pulses.

constant of the spike amplitude decay, and τ_{slow} is the slow time constant of the spike amplitude decay.

The parameters that yielded the best fit for the spike amplitude decay were calculated for all the cells measured. A summary of the distribution of the parameters that yield the best fit for the cells that were stimulated with 40Hz depolarizing pulses (similar to the one shown in Fig D) is shown in Table A.

In Table B, the summary of the distribution of parameters that yield the best fit for the traces stimulated with short hyperpolarizating pulses (similar to the trace shown in fig 5 in the main text) is shown.

The comparison of the distributions of parameters that yield the fist for the two different stimulation protocols is shown in Fig E.

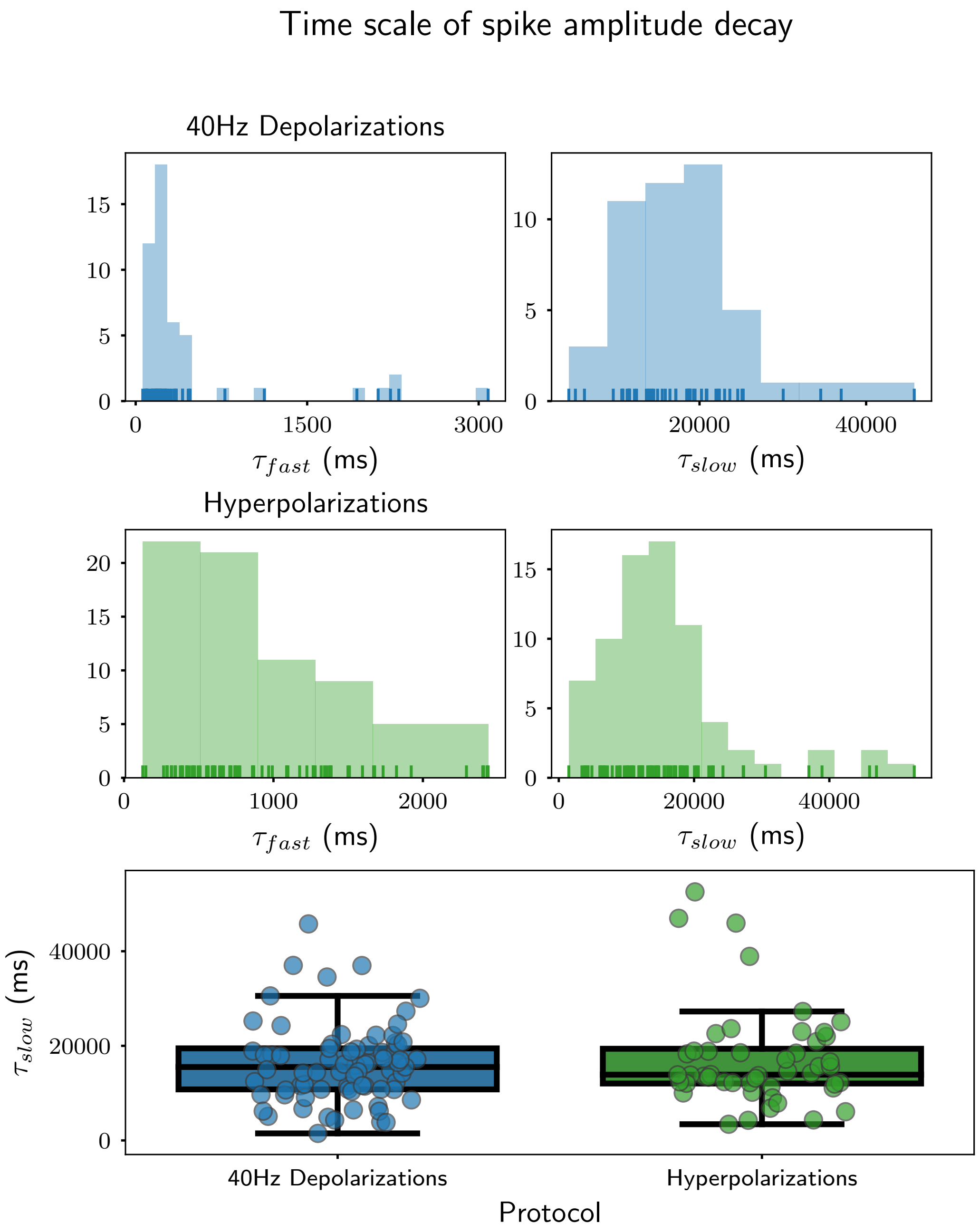

Fig E. Distribution of time scales of the double exponential decay (equation B) of the spike amplitude. Two protocols were used to measure the time scales of spike amplitude decay, an example of the "40 Hz Depolarizations" is shown in Fig. D, and an example of the "Hyperpolarization" is shown in Fig. 5 Notice that the distribution of τ_{slow} is independent of the protocol used.
