# Supplementary material for: Activity-mediated accumulation of potassium induces a switch in firing pattern and neuronal excitability type
Source: PLoS Comput Biol. 2021 May 27;17(5):e1008510. doi: 10.1371/journal.pcbi.1008510 (PMC8205125; doi:10.1371/journal.pcbi.1008510)
Supplement: S3 Text — An increase from 3mM to 12mM in extracellular potassium increased the spiking variability of 5 out of 6 cells measured. Fig G. Spiking variability calculated as the coefficient of variation (CV=σμ) for all cells sampled, when stimulating with baseline input. An increase from 3mM to 12mM in extracellular potassium increased the spiking variability of 2 out of 2 cells measured. The main source of stimuli irregularity was the network activity. Fig H. Spiking variability calculated as the coefficient of variation (CV=σμ) for all cells sampled after blocking synaptic input, under baseline input stimulation. An increase from 3mM to 10mM in extracellular potassium increased the spiking variability of 8 out of 12 cells measured. Fig I Spiking variability calculated as the coefficient of variation (CV=σμ) for all cells sampled. An increase from 3mM to 10mM in extracellular potassium increased the spiking variability of 3 out of 10 cells measured. The main source of stimuli irregularity was the network activity. (PDF) [file pcbi.1008510.s004.pdf]

Activity-mediated accumulation of potassium induces a switch in firing pattern and neuronal excitability type

Susana Andrea Contreras[1,2], Jan-Hendrik Schleimer[1,2], Allan T. Gulledge[3], Susanne Schreiber*[1,2]

1 Institute for Theoretical Biology, Humboldt-University of Berlin, Berlin, Germany.

2 Bernstein Center for Computational Neuroscience Berlin, Berlin, Germany.

3 Molecular and Systems Biology, Geisel School of Medicine at Dartmouth College, Hanover, New Hampshire, United States.

*Corresponding Author Susanne Schreiber
E-mail: s.schreiber@hu-berlin.de

Supporting information

S3 Text: Spiking irregularity

As mentioned in the main text, spiking irregularity increased with increases of the extracellular potassium concentration. Current-induced activity in mouse cortical pyramidal neurons exposed to different fixed concentrations of extracellular potassium was recorded. Action potentials were induced by constant-current stimulation in baseline conditions (3 mM extracellular potassium), and after increasing the concentration of extracellular potassium to 10 or 12 mM (see Materials and methods section in the main text). Neurons were stimulated with somatic current injection sufficient to maintain the membrane potential close to spiking threshold (Fig 4 in the main text).

Spiking irregularity was quantified in all the neurons recorded with the coefficient of variation (CV);

$$CV = \frac{\sigma}{\mu}, \tag{C}$$

where σ is the standard deviation of the interspike interval (ISI), and μ is the mean.

The summary of the CV change for all the neurons recorded is shown in Figs F,G,H,and I.

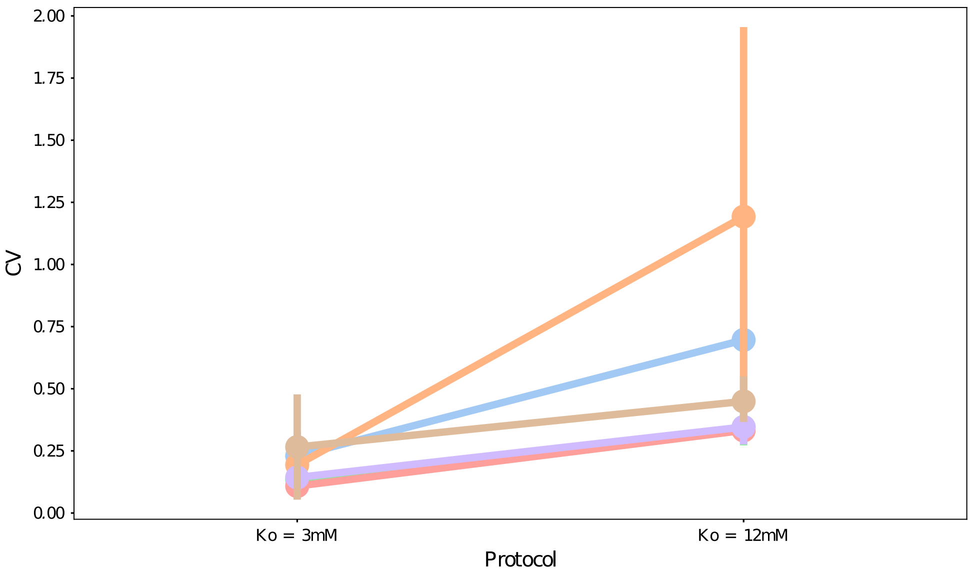

Fig F. Spiking variability calculated as the coefficient of variation ($CV = \frac{\sigma}{\mu}$) for all cells sampled, when stimulating with white noise added to the baseline input (n=6). An increase from 3 mM to 12 mM in extracellular potassium increased the spiking variability of 5 out of 6 cells measured.

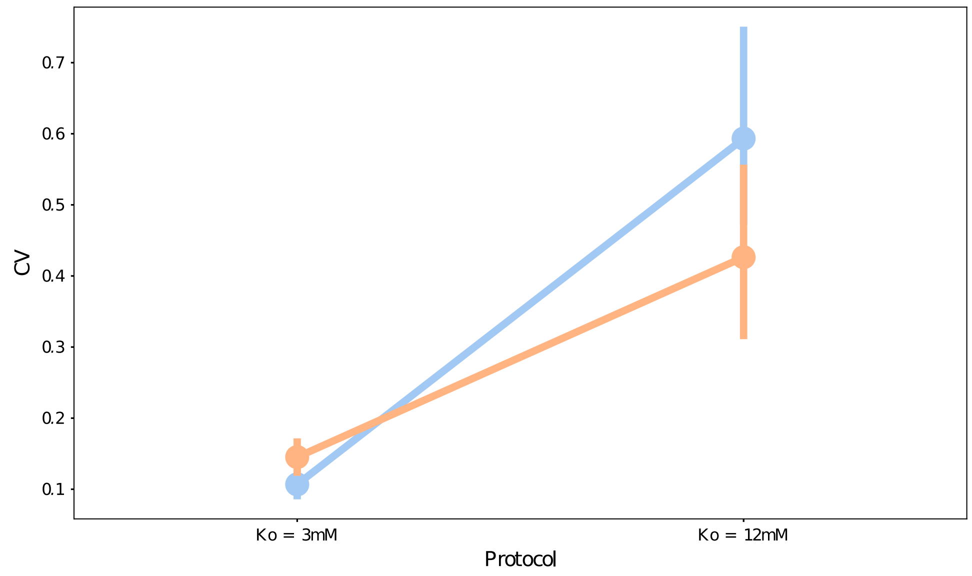

Fig G. Spiking variability calculated as the coefficient of variation ($CV = \frac{\sigma}{\mu}$) for all cells sampled, when stimulating with baseline input (n=2). An increase from 3 mM to 12 mM in extracellular potassium increased the spiking variability of 2 out of 2 cells measured. The main source of stimuli irregularity was the network activity.

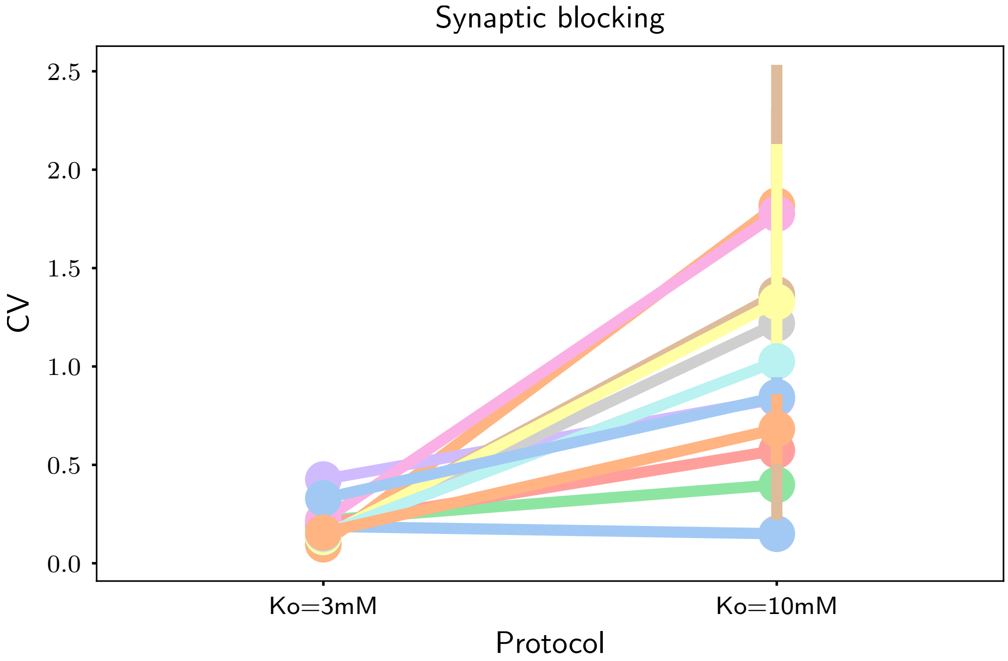

Fig H. Spiking variability calculated as the coefficient of variation ($CV = \frac{\sigma}{\mu}$) for all cells sampled after blocking synaptic input, under baseline input stimulation (n=12). An increase from 3 mM to 10 mM in extracellular potassium increased the spiking variability of 8 out of 12 cells measured.

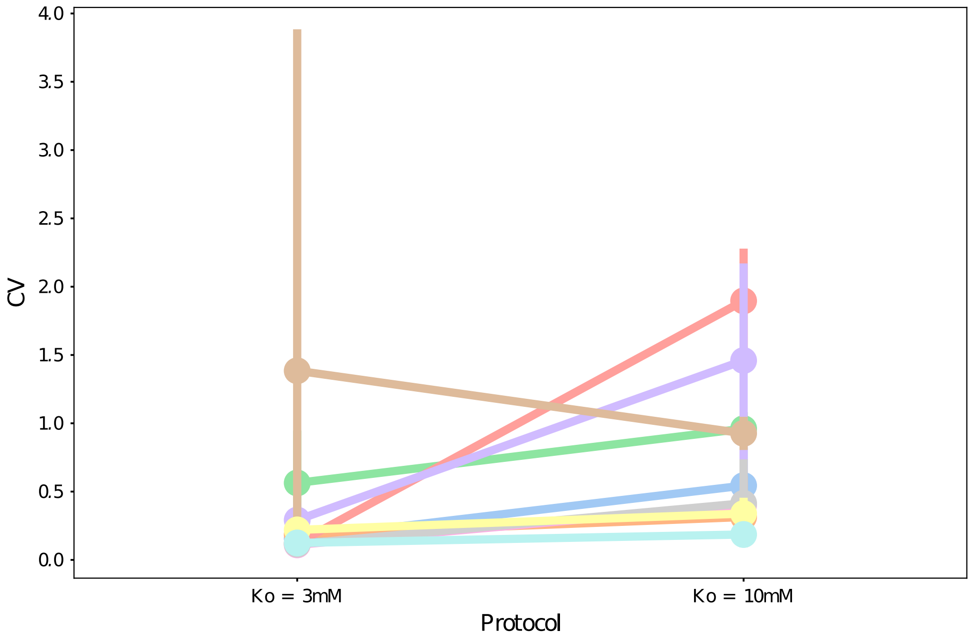

Fig I. Spiking variability calculated as the coefficient of variation ($CV = \frac{\sigma}{\mu}$) for all cells sampled (n=10). An increase from 3 mM to 10 mM in extracellular potassium increased the spiking variability of 3 out of 10 cells measured. The main source of stimuli irregularity was the network activity.
