# Supplementary material for: Activity-mediated accumulation of potassium induces a switch in firing pattern and neuronal excitability type
Source: PLoS Comput Biol. 2021 May 27;17(5):e1008510. doi: 10.1371/journal.pcbi.1008510 (PMC8205125; doi:10.1371/journal.pcbi.1008510)
Supplement: S4 Text — Table C. Gating dynamics used for the excitable portion of the model. Table D. Expressions used for the excitable portion of the model. Table E. Parameters used for the excitable portion of the model. Table F. Parameters used for the ionic concentration dynamics portion of the model. (PDF) [file pcbi.1008510.s005.pdf]

Activity-mediated accumulation of potassium induces a switch in firing pattern and neuronal excitability type

Susana Andrea Contreras[1,2], Jan-Hendrik Schleimer[1,2], Allan T. Gulledge[3], Susanne Schreiber*[1,2]

1 Institute for Theoretical Biology, Humboldt-University of Berlin, Berlin, Germany.

2 Bernstein Center for Computational Neuroscience Berlin, Berlin, Germany.

3 Molecular and Systems Biology, Geisel School of Medicine at Dartmouth College, Hanover, New Hampshire, United States.

*Corresponding Author Susanne Schreiber

E-mail: s.schreiber@hu-berlin.de

Supporting information

S4 Text: Model parameters and expressions

Gating dynamics	
$\frac{dm_{Na}}{dt}$	$\alpha_m (1 - m_{Na}) - \beta_m m_{Na}$
$\frac{dh_{Na}}{dt}$	$\alpha_h (1 - h_{Na}) - \beta_h h_{Na}$
$\frac{dn_K}{dt}$	$\alpha_n (1 - n_K) - \beta_n n_K$

Table C. Gating dynamics used for the excitable portion of the model.

Functions	
E_K	$\frac{RT}{F} \log \left(\frac{K_o}{K_i} \right)$
E_L	$\frac{RT}{F} \log \left(\frac{K_o P_K + Na_o P_{Na}}{K_i P_K + Na_i P_{Na}} \right)$
E_{Na}	$\frac{RT}{F} \log \left(\frac{Na_o}{Na_i} \right)$
α_h	$\frac{0.128 q_h e^{-\frac{\alpha_{hV}}{18} - \frac{v}{18mV}}}{ms}$
β_h	$\frac{4q_h}{ms\left(e^{-\frac{\beta_{hV}}{5} - \frac{v}{5mV}} + 1\right)}$
α_m	$\frac{q_m\left(0.32\alpha_{mV} + \frac{0.32v}{mV}\right)}{ms\left(1 - e^{-\frac{\alpha_{mV}}{4} - \frac{v}{4mV}}\right)}$
β_m	$\frac{q_m\left(0.28\beta_{mV} + \frac{0.28v}{mV}\right)}{ms\left(e^{\frac{\beta_{mV}}{5} + \frac{v}{5mV}} - 1\right)}$
β_n	$\frac{0.5 q_n e^{-\frac{\beta_{nV}}{40} - \frac{v}{40mV}}}{ms}$

Table D. Expressions used for the excitable portion of the model.

Parameters excitable portion	
g_K	$\frac{200mS}{cm^2}$
g_L	$\frac{0.1mS}{cm^2}$
g_{Na}	$\frac{100mS}{cm^2}$
C	$\frac{1.0uF}{cm^2}$
α_{hV}	50
α_{mV}	54
α_{nV}	52
β_{hV}	27
β_{mV}	27
β_{nV}	57

Table E. Parameters used for the excitable portion of the model.

Parameters ionic concentration dynamics	
ρ	$\frac{4000}{cm}$
F	$\frac{96484.6C}{mol}$
$\frac{Vol_i}{Vol_e}$	0.2
I_{max}	$\frac{40.0uA}{cm_2}$
$[Na^+]_o$	$140mM$
Na_s	$\frac{0.1}{mM}$
K_{Na}	$20mM$

Table F. Parameters used for the ionic concentration dynamics portion of the model.
