# Supplementary material for: Activity-mediated accumulation of potassium induces a switch in firing pattern and neuronal excitability type
Source: PLoS Comput Biol. 2021 May 27;17(5):e1008510. doi: 10.1371/journal.pcbi.1008510 (PMC8205125; doi:10.1371/journal.pcbi.1008510)
Supplement: Attachment — Submitted filename: ResponseToReviewers.pdf [file pcbi.1008510.s006.pdf]

Statement on the Revision of ⟨PCOMPBIOL-D-20-02004⟩ Based on the Referees' Report

Susana Andrea Contreras   Jan-Hendrik Schleimer
Allan T. Gulledge   Susanne Schreiber

March 7, 2021

This statement concerns our revision of the ⟨PCOMPBIOL-D-20-02004⟩ paper, entitled "*Activity-mediated accumulation of potassium induces a switch in firing pattern and neuronal excitability type*", based on the referees' report.

Reviewer #1

> In this manuscript, Contreras et al. investigate how activity-dependent dynamics in the extracellular potassium and intracellular sodium concentrations affect the dynamics of action potential generation in a computational model. This study highlights the importance of considering the often-overlooked role of ion plasticity in shaping neuronal spiking dynamics. The authors demonstrate that activity-induced short-term ionic dynamics can induce changes in firing mode, spike height, spike frequency adaptation, and excitability type. Additionally, the authors provide experimental evidence supporting their findings. Overall, I am enthusiastic about this work but have some concerns.

Major concerns

> `The leak current conducts both Na+ and K+ ions. Therefore, in Eq. 7 only a portion of IL should contribute to potassium concentration dynamics. Since IL conducts IK the leak reversal potential should also be dynamic, however, it is not clear from the main text if this is the case.`

The expression for the Nernst potential does include the sodium and potassium concentrations and varies with the ionic concentrations. Particularly we assume that the leak Nernst potential can be described with an approximation for the Goldman–Hodgkin–Katz (GHK) membrane potential. This has been clarified in the main text, lines 451 and 452.

> `The model of the Na-K-ATPase pump used here does not include any activation dependence on potassium or voltage. It should be discussed how more complicated pump activation dynamics might affect these findings.`

We used an analog to NaK-ATPase isoform α_3, which is mostly sensitive to intracellular sodium in the regime we studied. In the appendix we added Fig.S10 that illustrates how the quantitative behavior of the model would be distorted with a different pump isoform sensitive to extracellular potassium (resembling α_2). We also added a paragraph in the method section, lines 465-476, commenting on the fact that the pump choice quantitatively influences the results presented here.

> `The experimental data showing a switch from a regular to an intermittent firing pattern would be more convincing with additional neurons and if synaptic transmission were blocked.`

We thank the reviewer for the comment, we did follow the suggestion and recorded from slices with synaptic blockade while repeating the protocol to rule out that the source of irregularity came from network non-linearities (Fig. S8), and lines (210-212).

> The bifurcation analysis presented here clearly shows how increasing extracellular potassium leads to a bistable state by the creation and coexistence of a stable fixed point and a stable limit cycle. However, a biophysical explanation could make the findings accessible to a broader audience and provide additional intuition into the underlying dynamics. More specifically, how does depolarizing EK and EL (by increasing extracellular K+) affect the balance of INa, IK and IL resulting in the creation of a stable limit cycle without destroying the stable node?

We added an intuition into the discussion (lines 321-329) which reads;

"In this particular setting, an increase in the Nernst potential for potassium (E_K) and the Nernst potential for the leak (E_L) by constraining the magnitude of the potassium (I_K) and leak (I_L) currents, respectively, shift the spike (limit cycle orbit) towards more depolarized membrane potentials and farther from the resting state (stable node). When both attractors are sufficiently far from each other, both can coexist. The stable node does not loose its stability due to the hyperpolarizing current that is received by the neuron model, either via an external input (Fig.3), or via the hyperpolarizing pump current (Fig.6). Interestingly, other parameters such as temperature and capacitance can also induce the same bistability reported here Hesse et al. [2017] via a different biophysical mechanism yet identical bifurcation structure."

> Similarly, how robust is the bistable region presented in Fig. 3A and Fig. 6 to changes in model parameters? For example, if g_K and/or g_L is increased/decreased does this affect the size and location of the bistable region? Manipulation of extracellular potassium ultimately affects the magnitude of IK and IL. Therefore, g_K and g_L should affect the bistable region.

This is a good point, indeed g_K, and g_L distort the bistable region, because by increasing or decreasing the amplitude of their respective currents, they do shift the saddle node bifurcation location, as well as the limit cycle onset. We added Figs. S2 and S3 in the appendix to illustrate how the g_K, and the g_L conductances distort the bistable region. We also added a reference to Fig.S3 in the discussion to clarify that indeed the bistable region can be distorted (line 381-383).

Minor concerns

> In the model spike frequency adaptation occurs due to Na+ accumulation and the subsequent outward current generated by the Na/K pump. Typically, increasing extracellular K+ increases excitability and firing rate by depolarizing EL and EK. Would spike-frequency adaptation still occur if gL and/or gK were larger?

Spike frequency adaptation could be diminished by a lower initial firing and/or a more efficient action potential shape. g_L and g_K do alter the initial firing rate and the limit cycle shape, thus the magnitude of the spike-frequency adaptation is altered, but not abolished (see Fig. 1).

> The orange trajectory in Fig. 7A is said to reach the bistable region, however it looks like the full trajectory remains in the green spiking region.

We thank the reviewer for the comment, we zoomed-in to improve the figure's resolution.

> References to supplemental figures do not have figure numbers. See line 197, 224 and 238 for example.

We thank the reviewer for the comment, the references to the supplemental figures were added.

> Line 368 is missing "of" at the end of the sentence between "effects" and "inactivation".

We thank the reviewer for the correction, the main text was modified accordingly.

> Eq. 5 label should be right justified like the rest of the equations.

Thank you, the label has been fixed in the main text.

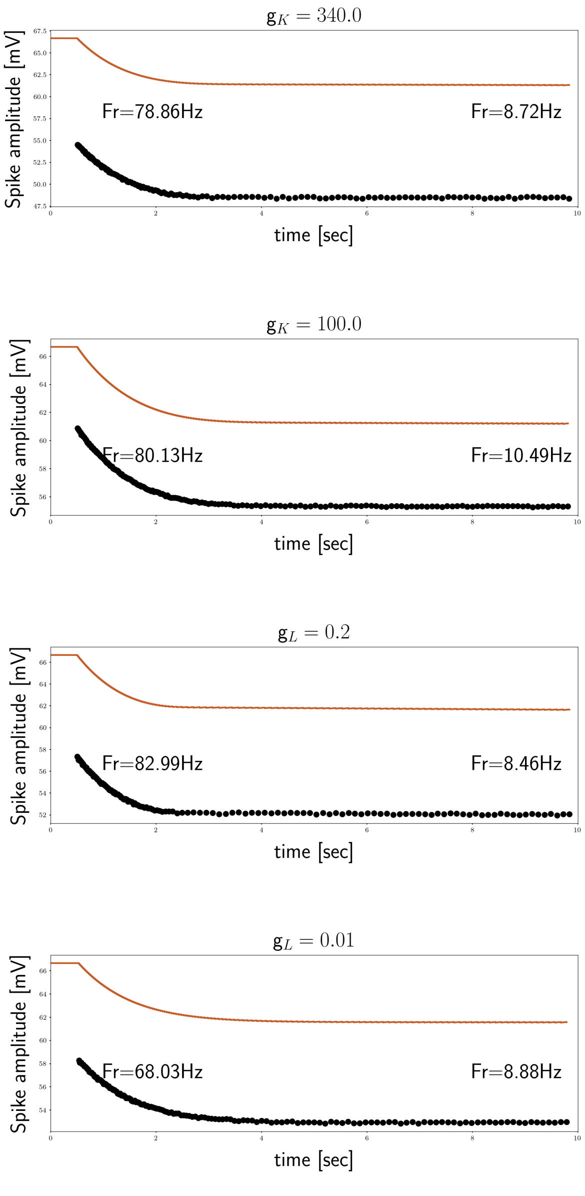

Figure 1: Spike frequency adaptation for different conductances (g_K and g_L).

Reviewer #2

> The authors present a single compartment neural model, including standard ion channels (Na+ K+) for AP generation, leak currents and a Na/K-pump. They model the ion concentrations for Na+ (intracellularly) and K+ (intra- and extracellularly). The modeling work seems appropriately conducted. As the main frontier of the computational neuroscience field (HBP and so) typically neglects effects of ion concentration dynamics altogether, I am happy to see models that do not. The model is used to study the consequences that varying ion concentrations has on the firing property of neurons. Some of the key findings are validated through experiments. The perhaps most interesting finding is that extracellular potassium accumulation switches the neuron into a bistable regime described through a to a homoclinic orbit bifurcation (HOM), where it produces intermittently interrupted (burst-like) output.

Major concerns

Although, as mentioned, the standard within the field is to neglect effects of varying ion concentration altogether, there is quite a number of computational works addressing the topic, often in simple single compartment neuron models such as the one presented here. Some papers that seems to be close in topic to the here submitted work are:

- Kager, H., Wadman, W. J., Somjen, G. G. (2000). Simulated seizures and spreading depression in a neuron model incorporating interstitial space and ion concentrations. Journal of neurophysiology, 84(1), 495-512.
- Cressman, John R., et al. "The influence of sodium and potassium dynamics on excitability, seizures, and the stability of persistent states: I. Single neuron dynamics." Journal of computational neuroscience 26.2 (2009): 159-170.
- Øyehaug, L., Østby, I., Lloyd, C. M., Omholt, S. W., Einevoll, G. T. (2012). Dependence of spontaneous neuronal firing and depolarisation block on astroglial membrane transport mechanisms. Journal of computational neuroscience, 32(1), 147-165.
- Owen, J. A., Barreto, E., Cressman, J. R. (2013). Controlling seizure-like events by perturbing ion concentration dynamics with periodic stimulation. PLoS One, 8(9), e73820
- Hübel, N., Schöll, E., Dahlem, M. A. (2014). Bistable dynamics underlying excitability of ion homeostasis in neuron models. PLoS Comput Biol, 10(5), e1003551.
- Wei, Y., Ullah, G., Schiff, S. J. (2014). Unification of neuronal spikes, seizures, and spreading depression. Journal of Neuroscience, 34(35), 11733-11743
- Sætra, M. J., Einevoll, G. T., Halnes, G. (2020). An electrodiffusive, ion conserving Pinsky-Rinzel model with homeostatic mechanisms. PLoS computational biology, 16(4), e1007661.

> I was surprised to see that neither of these were cited in the manuscript, and I am a bit uncertain as to how the current paper and its conclusions stand out as compared to these previous works. For example, the works by Cresman et al (2009), Øyehaug et al. (2012), Hübel et al (2014) and Wei et al. (2014) all present some bifurcation analysis relating neuronal firing properties to some model variables or parameters, and extracellular K+ play an important role in most of these. Probably, there is something new to the kind of bistability observed in the submitted manuscript relative to the different firing regimes reported previously, but I think a comparison should be made in this regard.

We thank the reviewer for pointing out the relevance of these articles.

Particularly in the studies of Cressman et al. [2009], Øyehaug et al. [2012], Wei et al. [2014b] and Owen et al. [2013], the slow and the fast dynamics are separated as in our work. In Hübel et al. [2014] the complete model (excitable neuron with ionic concentration dynamics) is simplified, by eliminating the very fast time scale (sodium activation), and making the sodium inactivation a function of the potassium inactivation. Unlike our work, these articles [Cressman et al., 2009, Øyehaug et al., 2012, Wei et al., 2014b, Hübel et al., 2014, Owen et al., 2013] mainly focus on the slow ionic concentration dynamics and their equilibrium states. We, on the other hand, focus on the transient effect of ionic concentration dynamics on neuronal excitability. Although some of the articles the reviewer mentioned (Kager et al. [2000], Sætra et al. [2020]) also focus on transient effects, in those the neuron is stimulated with static input which doesn't allow the detection of the stochastic bursting reported in this work.

- Cressman et al. [2009] analyze a model with a fast spike generating system similar to the one proposed here, however, they include a calcium-gated potassium current and a chloride dependent leak. In their model, Cressman et al. [2009], do account for potassium diffusion throughout the extracellular space (or vasculature in the intact brain) and glial buffering unlike we do in this work. The equilibrium states of the model reported by Cressman et al. [2009] are deduced from a bifurcation analysis performed on the slow dynamics only. The authors find that by increasing the extracellular potassium concentrations on the bath (in a slice or the potassium concentration in the vasculature in the intact brain), the extracellular potassium concentration in the local spatial vicinity of the neuron could go from a constant value (steady state) to an oscillation (stable limit cycle) and when increased further to a higher constant value (steady state). The authors also show how altering the homeostatic mechanisms that control the extracellular potassium concentration (Na-K-ATPase pump strength, diffusion coefficient, and glial strength) may reduce or amplify the extracellular potassium concentration oscillation once it occurred. In their work,

the authors also include example traces of the membrane potential during the mentioned extracellular potassium concentration oscillations. Those traces are characterized by deterministic bursting or in other words a fixed inter burst interval. The deterministic bursting is generated by the slow oscillation of the extracellular potassium concentration that forces the fast subsystem to undergo a bifurcation from a silent (steady state) to an excited (stable limit cycle). In order to perform the bifurcation analysis of the concentration dynamics, the authors average the contribution of the fast spike generating currents after reaching a steady state (or asymptotic state). This type of analysis assumes a constant input.

The work presented here doesn't include a detailed description of the homeostatic mechanisms for the extracellular potassium concentration as Cressman et al. [2009] exposed, instead, here we focus on the transient effect that this ionic concentration change has in the fast subsystem accounting for the fact that synaptic input, due to its biophysical nature, is never completely constant. Our simulations show bursting as well, however, inter-burst intervals are not constant given that the bursts reported here arise from the bistability that emerges in the fast spike generating dynamics when extracellular potassium concentration increases.

- Øyehaug et al. [2012] analyze a single compartment neuron-model similar to the one presented here, plus a persistent sodium current, and a transient potassium current. The authors include a glial cell-model that regulates the ionic concentration dynamics in the extracellular space. Øyehaug et al. [2012] also perform a time scale separation between the fast spike generating dynamics, and the slow concentration dynamics. They, very thoroughly analyse the dynamics of both the fast and the slow system. For the fast spike generating system, a bifurcation with respect to extracellular potassium and the intra-neuronal sodium is presented. It is worth highlighting that they report a bistable region delimited by the same bifurcations reported here (saddle-node, homoclinic, and hopf bifurcations). The bistable region is located at slightly different extracellular potassium and intracellular sodium concentrations in their model, but as mentioned in the main text, this is not surprising given that some parameters (like the leak conductance, the capacitance and the temperature) could shift the location of the bistable region. Given that Øyehaug et al. [2012] focus on understanding how ionic homeostatic mechanisms can alter the dynamics of extracellular potassium, the authors don't discuss the implications of observing the extracellular potassium concentration dependent bistable region of the fast spike generating dynamics. The input of their model is constant, which explains why in the membrane potential traces they portray, consequently no irregular bursting is present. As well as Cressman et al. [2009] do, Øyehaug et al. [2012] report deterministic bursting caused by the oscillation of extracellular potassium and chloride concentrations.

- Wei et al. [2014b] analyze a model with fast spike generating dynamics

identical to the ones proposed here. However, the description of the homeostatic mechanisms that control extracellular concentrations include lateral extracellular potassium diffusion and glial buffering. The authors perform a time scale separation, and analyse the dynamics of the fast and the slow system. The change of the spike generating dynamics is shown relative to changes in the bath potassium concentration. In their bifurcation diagram the dynamical system goes from a steady state, to a stable limit cycle as the bath potassium concentration increases (through a saddle node on invariant circle bifurcation or SNIC). Further increases of the potassium bath concentration push the fast dynamics to a depolarized steady state through a hopf bifurcation. Wei et al. [2014a] don't show the bifurcation diagram of the fast spike generating mechanism relative to extracellular potassium concentration (which differs from the potassium bath concentration), thus, we can not state whether the homeostatic mechanisms in their model would prevent the fast spike generating dynamics to display the bistable dynamics reported in our work. Similarly to Cressman et al. [2009] and Øyehaug et al. [2012]; Wei et al. [2014b], perform a bifurcation analysis of the slow ionic concentration dynamics relative to the bath potassium concentration, but go a step forward by comparing the simplified Hugking-Huxley formulation with the Goldman–Hodgkin–Katz (GHK) formulation for all the spike generating currents. Qualitatively both are similar, however, the exact locations of the bifurcations differ.

What Cressman et al. [2009], Øyehaug et al. [2012], and Wei et al. [2014b] have in common is that they all report limit cycles on the slow ionic concentration dynamics. Particularly, the extracellular potassium concentration. Given that extracellular potassium determines how excitable is the neuron, in those models the constant oscillation of the concentration pushes the neuron-model from a silent state (stable node) to an excited state (stable limit cycle). They all report ionic concentration oscilations with different frequency ranges (0.01 to 0.25 Hz), and steady vs. oscillatory equilibria occurring at different bath potassium concentrations. Probably all are plausible in the brain given different physiological or pathophysiological states, however, more experimental evidence regarding ionic dynamics is needed to characterize in detail which model fits which state.

Given the ample range of studies of the slow ionic concentration dynamics, here, we chose to zoom in and focus on the transient ionic dynamics before reaching an equilibrium, zooming-in the detail to understand the mutual effect of the slow ionic concentration dynamics on the fast spike generating dynamics and vice-versa with static and fluctuating input.

We added the following lines (16-24) to the introduction to contextualize our work properly

"A number of simulation studies have analysed the slow ionic concentration dynamics and their equilibria Kager et al. [2000], Cressman et al. [2009], Øyehaug et al. [2012], Wei et al. [2014b], Hübel et al. [2014], portraying mechanistic explanations of the emergence of slow ionic concentration oscillations (0.5-10

cycles per minute). These studies have particularly focused on understanding how ionic homeostasis Øyehaug et al. [2012], Wei et al. [2014a], Hübel et al. [2014], Cressman et al. [2009] or stimulus properties Owen et al. [2013] may shape ionic concentration equilibria. However, only few have analysed the transient effect of these concentration changes on neuronal excitability Kager et al. [2000, 2007]. The majority of the aforementioned studies have adopted a static stimulation, except for Owen et al. [2013] who showed that a periodic step current injection can drastically alter ionic concentration equilibria."

We also added citations in the discussion (line 337).

Regarding the model

> `I do not have the book where the Traub-Miles model (used in the current work) is described. I would like to ask why this model was selected for the study. As far as I understand, it is supposed to represent a hippocampal neuron, while the experimental validation was based on a cortical neuron.`

We chose the Traub-Miles model because it is one of the simplest mammalian models that display type I excitability. Neurons with type I excitability have a continuous and smooth FI curve, thus, display very low firing rates like cortical neurons do. The type I assumption is fundamental to our analysis, while the exact currents of the model are less relevant.

We added the justification to the method section (lines 444-445).

> `Do you think that your conclusions regarding the bistability regime is sensitive to the choice of model, or do you picture it to be of general validity, i.e., be true for, say, the Hodgkin-Huxley model or any other existing model including the standard Na+ and K+ channels.`

Our conclusions depend mainly on the excitability type of the neuron model at the onset of stimulation. The model must exhibit type I dynamics, i.e be capable of transitioning smoothly from silent states to low firing rates as the input current is increased. (We added line 33 to the main text to make this precondition clear).

The majority of slow firing conductance based models, transition smoothly from a silent state to an excited state through a SNIC (saddle node on invariant cycle) bifurcation. These models display an S-shaped (V-I_{app}) curve (Ermentrout and Terman [2010], Chapter 6.2), resulting in a system with three fixpoints. (We included this intuition in lines 126-127).

In the original Hodgkin and Huxley [1952] model the bistability we report here is possible, but with very high extracellular potassium concentrations. Then the projection of the fixed points on the V-I_{app} plane curves resembles an S-shape

(see Fig.2). However, the system first had to undergo a CUSP bifurcation to switch from one to threee fixpoints. The extracellular potassium concentration range in which this occurs is far from the physiological range, notice that the curve folds only at positive EK.

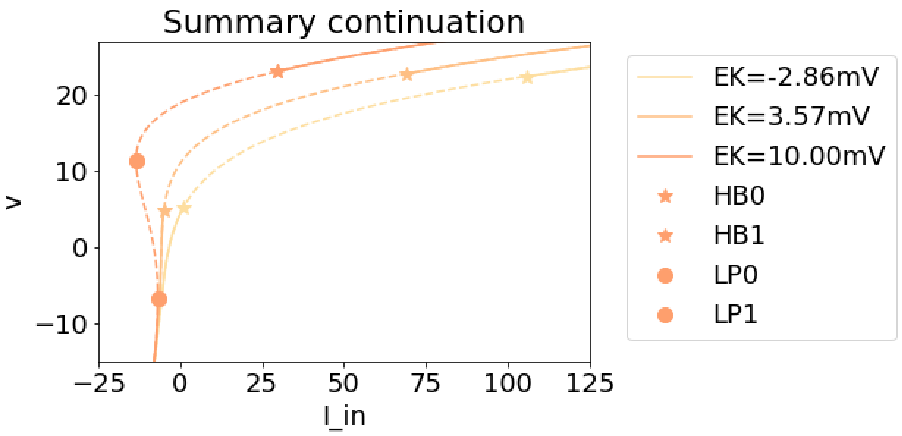

Figure 2: Fixed points of the original Hodgkin and Huxley [1952] model

The transition through the CUSP might occur in other models as well, but there is not yet a generalized formal proof that would guarantee it.

Other reported instances of the extracellular potassium dependent bistability reported here can be found in:

- Barreto and Cressman [2011], analysed the qualitative behavior of a modified Hodgkin-Huxley model. The bifurcation graph shown (in Fig. 5 in their article) implies a region of bistability bounded by the same bifurcations of the bistable region presented in our work, the saddle node, homoclinic, and hopf bifurcation lines.
- Øyehaug et al. [2012], analysed a model proposed by Kager et al. [2000], show the existence of a bistable region relative to $[K^{+}]_o$ and $[Na^{+}]_i$ (Fig.3 in their article) bounded by the same bifurcations mentioned above.
- Aihara and Matsumoto [1983], made a similar analysis for the original Hodgkin-Huxley model (relative to $[K^{+}]_o$ only) and found two possible bistable regimes. One in which the limit cycle onset is the HOM bifurcation like the one reported here (corresponding to the fixpoints curve with very positive EK in Fig.2), and the other one, in which the limit cycle onset is the Hopf bifurcation. The one that corresponds to our study (HOM) occurs at unrealistically high $[K^{+}]_o$. However, the bistability occuring in the Hodgkin-Huxley model for physiologically plausible concentrations (Hopf limit cycle onset), would also favor irregular interspike intervals when

exposed to irregular input with some differences in the interspike intervals [Schleimer et al., 2020].

We added the previous references to line 320 in the main text.

> As indicated by the formulation (line 422-424) \Two ingredients are needed: an excitable system (capable of generating spikes) for which we use the Traub-Miles formulation (See equations (1),(2),(3),(4)), and a description for the ionic concentration changes that occur due to ionic currents", the original Traub-Miller model did not include ion concentration dynamics. Hence, some of the parameters in Table S5 were not from the Traub-Miller model, but new to this paper. I think, then, that it would be appropriate to comment on how these were chosen.

We added a reference and an explanation for the parameter choices in lines 457-459, and lines 479-486.

> The values for the parameters Vol_i and Vol_e (or the ratio between them) were not given in the paper.

We thank the reviewer for pointing this out. The table.S6 was fixed to include those values, and lines 482-484 where added to justify the choice.

> As in most models of this kind, the extracellular space was confined, and K+ would accumulate locally without being allowed to "diffuse" away. I suppose it could be added to the discussion that this is equivalent to having periodic boundary conditions (if all neurons in the neighborhood do the same, all extracellular space receive the same amount of K+, so there will be no concentration gradients, and thus no extracellular diffusion).

We thank the reviewer for pointing this out, we added a comment highlighting the fact in lines 413-417.

> The parameter rho had the value 4/cm. Since \rho/F" converts a membrane current density to an intracellular concentration change, rho should interpret as the surface to volume ratio of the neuron. Picturing a spherical neuron, that would give us: $rho = 4\pi r^2/(4/3\pi r^3) = 3/r$. For rho to have the value 4/cm, r must be $3/4cm$, which is a very huge neuron. Any comments on that?

We thank the reviewer for pointing this out. There was a unit mismatch in the parameter table, we corrected this (table S.6), and added some references in the main text to support our parameter choices.

Regarding the presentation

> I would like to find a summarizing figure in the beginning of the results section that illustrates everything that goes into the model (the included membrane mechanisms and key variables, Vm, Na+ and K+).

We have followed the suggestion of the reviewer, and included Fig.8 in the methods section.

> The model should be made available online.

We followed the suggestion of the reviewer, the model can be found in the following repository.

Reviewer #3

> The authors present a neuronal model with standard HH conductances with ionic dynamics including an electrogenic pump. The authors perform a fast slow analysis to investigate the role of ionic dynamics in the behavior of a single neuron and identify the dynamical structure underlying a number of possible neuronal states. The work is interesting and the results are promising, but there are a number of issues that need to be addressed before consideration for publication.

Major concerns

> The fast slow analysis described here is similar to previous work by Owen et al (2013) Controlling Seizure-Like Events by Perturbing Ion Concentration Dynamics with Periodic Stimulation. PLOS ONE 8(9): e73820. The role of sodium and potassium dynamics in setting the state of the cell is described in that work. The relation to this work should be addressed.

We thank the reviewer for pointing out the relevance of this article to our work.

The following paragraph has been added to the introduction (16-24):

"A number of simulation studies have analysed the slow ionic concentration dynamics and their equilibria Kager et al. [2000], Cressman et al. [2009], Øyehaug et al. [2012], Wei et al. [2014b], Hübel et al. [2014], portraying mechanistic explanations of the emergence of slow ionic concentration oscillations (0.5-10 cycles per minute). These studies have particularly focused on understanding how ionic homeostasis Øyehaug et al. [2012], Wei et al. [2014a], Hübel et al. [2014], Cressman et al. [2009] or stimulus properties Owen et al. [2013] may shape ionic concentration equilibria. However, only few have analysed the transient effect of these concentration changes on neuronal excitability Kager et al. [2000, 2007]. The majority of the aforementioned studies have adopted a static stimulation, except for Owen et al. [2013] who showed that a periodic step current injection can drastically alter ionic concentration equilibria."

> One interesting effect described by the author is the multi-stable dynamics supported by their model. This multi-stability and switching is a manifestation of the well known inverse stochastic resonance. Please see this reference for details. Uzuntarla et al. Dynamical structure underlying inverse stochastic resonance and its implications Phys. Rev. E 88, 042712 { Published 31 October 2013. This point should be addressed.

We added a paragraph to the discussion addressing this point (lines 330-335), it reads:

"In the presence of a fluctuating input (be it noise or signal), the bistability renders neurons susceptible to switches between the two stable states, giving rise to an irregular, intermittently interrupted firing pattern of short firing phases and pauses of different durations. It has been shown that combining multistable systems, similar to the one reported here, with noise can have unexpected information transmission consequences like "inverse stochastic resonance" Uzuntarla et al. [2013], as well as drastic spiking statistics alterations Schleimer et al. [2020]. "

> The bi-stable state however is not convincingly motivated by the experimental results.

We blocked synaptic input and repeated the protocol to rule out that the source of irregularity comes from network non-linearities (Fig. S8), and lines (210-212).

> ```
> The potassium concentrations used are very high and
> one might expect seizures.  Under these conditions
> experimentally, (Jensen and Yaari.  1997, Role of intrinsic
> burst firing, potassium accumulation, and electrical
> coupling in the elevated potassium model of hippocampal
> epilepsy.  J. Neurophys.),
> ```

We thank the reviewer for pointing out this relevant experimental observation that aligns with our predictions. We have mentioned their work in the discussion (line 363).

One of the predictions of our analysis is that non-bursting neurons can display bursting-like activity due to extracellular potassium concentration changes, which aligns with the observations of Jensen and Yaari [1997].

Given that we don't include extracellular potassium regulatory mechanisms we can not reproduce the extracellular potassium oscillation that Jensen and Yaari [1997] report, which probably has a big influence on the burst termination, and the duration of the hyper-excitable state.

> ```
> and computationally ( Cressman and Ullah et al.  The
> influence of sodium and potassium dynamics on excitability,
> seizures, and the stability of persistent states:  I  II.
> Network and glial dynamics.  J. Computational Neuroscience.
> 2009.)  , using a similar method to what is performed here.
> These computational models also demonstrated multi stability
> due to ionic dynamics.  How do your results relate to these
> previous findings?
> ```

The following paragraph has been added to contextualize our work relative to theirs (Cressman et al. [2009]) (lines 16-24):

"A number of simulation studies have analysed the slow ionic concentration dynamics and their equilibria Kager et al. [2000], Cressman et al. [2009], Øyehaug et al. [2012], Wei et al. [2014b], Hübel et al. [2014], portraying mechanistic explanations of the emergence of slow ionic concentration oscillations (0.5-10 cycles per minute). These studies have particularly focused on understanding how ionic homeostasis Øyehaug et al. [2012], Wei et al. [2014a], Hübel et al. [2014], Cressman et al. [2009] or stimulus properties Owen et al. [2013] may shape ionic concentration equilibria. However, only few have analysed the transient effect of these concentration changes on neuronal excitability Kager et al. [2000, 2007]. The majority of the aforementioned studies have adopted a static stimulation, except for Owen et al. [2013] who showed that a periodic step current injection can drastically alter ionic concentration equilibria."

> I am not convinced that 2 out of 2 cells having a higher variability without blocking synapses with high potassium demonstrates an intrinsic cellular response.

We observed the effect in 7 out of 8 neurons tested, when potassium levels were increased from 3 mM to 12 mM (Figs. S6 and S7). When increasing the extracellular potassium concentration from 3 to 12 mM, 3 out of 10 neurons tested, showed an increase in spiking irregularity (Fig. S9).

And for this updated version of our work, we blocked synaptic input and repeated the protocol to rule out that the source of irregularity comes from network non-linearities (Fig. S7), and lines (210-213).

> Line 72 Why α 3? This seems like a strange pump to choose, and I would expect it to play a key role in the sodium potassium dynamics of the cell. This choice needs to be better motivated.

We added a motivation of our pump choice in the method section (lines 465-476). Which reads:

"Other modelling studies use pump models that resemble more closely Na-K-ATPase $\alpha 2$ isoform Owen et al. [2013], Wei et al. [2014a], Kager et al. [2000]. The main difference between both isoforms is that $\alpha 2$ has a much higher sensitivity to extracellular potassium and membrane potential than $\alpha 3$ isoform Clausen et al. [2017]. Developmental studies of isoform expression in the brain have shown that neuronal cells from newborn mice express mainly $\alpha 2$, whereas adult animals express mainly $\alpha 3$ Moseley et al. [2002]. Given that here experiments were performed in neurons of adult mice, the $\alpha 3$ isoform seems more appropriate.

To understand how our pump choice determines the qualitative results presented here, we performed a bifurcation analysis to the model described above with a pump expression that resembles pump isoform $\alpha 2$, refer to Fig.S7. The main difference between the pump expression in eq.5 and the one used to resemble $\alpha 2$ (Fig.S7), is the pump sensitivity to extracellular potassium. Fig.S7 illustrates how the pump sensitivity to $[K^+]_o$ shifts the spiking threshold towards higher input currents as $[K^+]_o$ accumulates."

> Line 78. This effect has been seen before with other models with ionic dynamics. Somjen et al Kager, et al, 2007,Seizure-like after discharges simulated in a model neuron. J. of Comp. Neuro)

Thank you for pointing out their work. Probably the reason why the model by Kager et al. [2007] and our model predict different sources of the spike amplitude

reduction is the duration of the stimulation. We speculate that if the model used by Kager et al. [2007] was exposed to longer stimuli, the portion of the spike amplitude reduction due to the intracellular sodium accumulation would increase.

We have included references to this work in the main text (lines 31,87,240).

Minor concerns

> `Line 61 slow AHP resulted from the hyperpolarizing Na-K-ATPase pump current: Did you check to see if the current was responsible for the hyperpolarization, or just the sodium driven decrease in potassium concentrations? Your pump is not sensitive to potassium so it could get very low outside driving your Ek and Vm very negative.`

Yes, we checked. And other experimental observations suggest this as well Gulledge et al. [2013].

> `Your pump is not sensitive to potassium so it could get very low outside driving your Ek and Vm very negative.`

Given that the leak current is dependent on potassium, it always tends to increase outside.

> `Line 134. Typo`

We thank the reviewer for the correction. This was fixed in the main text.

> `Line 135, permanently probably should be continuously.`

We thank the reviewer for the suggestion. This phrasing was changed in the main text.

> `Section starting on line 149. How were the different bifurcations identified?`

We added the following lines to the method section (lines 503-532):

"First a particular combination of ionic concentrations is chosen and the steady states of the dynamical system with the combination of parameters are calculated with a regular ODE, secondly the applied current is used as the continuation parameter. AUTO, detects the stable/unstable nodes and limit cycles, it also points where the stability of such objects changes (bifurcations). For this work the most relevant ones are; the points where the stable node transitions from

being stable to unstable (saddle-node bifurcation), the point where a stable limit cycle orbit emerges (HOM), and the point where the stable limit cycle disappears (Hopf bifurcation). Such points can be continued along all the model parameters efficiently in AUTO. Fig.6 is constructed by continuing the 3 points mentioned above, in the extracellular potassium - applied current plane."

We also made the code available in the following repository.

> `Figure 2. The resting state is below the potassium reversal for the quiescent state. This indicates some tonic negative input. What is the source of this input?`

The Na-K-ATPase is hyperpolarizing the neuron, due to the previous Na accumulation. The negative input that results from this current, consequently shifts the resting state towards more hyperpolarized membrane potentials than the potassium reversal potential.

> `Line 504, seg->ms`

We thank the reviewer for the correction, the text was edited from seg to sec. To highlight the scale difference between the slow and the fast time scale, both sec and ms are used.

> `Line 508, figure number missing`

We thank the reviewer for the correction. This was fixed in the main text.

References

References

K. Aihara and G. Matsumoto. Two stable steady states in the Hodgkin-Huxley axons. *Biophysical journal*, 41(1):87–9, 1 1983. ISSN 0006-3495. doi: 10.1016/S0006-3495(83)84408-7. URL `http://www.ncbi.nlm.nih.gov/pubmed/6824756http://www.pubmedcentral.nih.gov/articlerender.fcgi?artid=PMC1329016`.

E. Barreto and J. R. Cressman. Ion concentration dynamics as a mechanism for neuronal bursting. *Journal of Biological Physics*, 37(3):361–373, 6 2011. ISSN 0092-0606. doi: 10.1007/s10867-010-9212-6. URL `http://www.ncbi.nlm.nih.gov/pmc/articles/PMC3101327/`.

M. V. Clausen, F. Hilbers, and H. Poulsen. The Structure and Function of the Na,K-ATPase Isoforms in Health and Disease. *Frontiers in Physiology*, 8: 371, 6 2017. ISSN 1664-042X. doi: 10.3389/fphys.2017.00371. URL http://journal.frontiersin.org/article/10.3389/fphys.2017.00371/full.

J. R. Cressman, G. Ullah, J. Ziburkus, S. J. Schiff, and E. Barreto. The influence of sodium and potassium dynamics on excitability, seizures, and the stability of persistent states: I. Single neuron dynamics. *Journal of Computational Neuroscience*, 26(2), 2009. ISSN 09295313. doi: 10.1007/s10827-008-0132-4.

G. B. Ermentrout and D. H. Terman. Mathematical foundations of neuroscience. In *Interdisciplinary Applied Mathematics*, volume 35. 2010.

A. T. Gulledge, S. Dasari, K. Onoue, E. K. Stephens, J. M. Hasse, and D. Avesar. A sodium-pump-mediated afterhyperpolarization in pyramidal neurons. *The Journal of Neuroscience*, 33(32):13025–13041, 8 2013. doi: 10.1523/JNEUROSCI.0220-13.2013.

J. Hesse, J.-H. Schleimer, and S. Schreiber. Qualitative changes in phase-response curve and synchronization at the saddle-node-loop bifurcation. *PHYSICAL REVIEW E*, 95, 2017. doi: 10.1103/PhysRevE.95.052203. URL https://journals.aps.org/pre/pdf/10.1103/PhysRevE.95.052203.

A. L. Hodgkin and A. F. Huxley. A quantitative description of membrane current and its application to conduction and excitation in nerve. *The Journal of Physiology*, 117(4):500–544, 8 1952. ISSN 00223751. doi: 10.1113/jphysiol.1952.sp004764. URL http://doi.wiley.com/10.1113/jphysiol.1952.sp004764.

N. Hübel, E. Schöll, and M. A. Dahlem. Bistable Dynamics Underlying Excitability of Ion Homeostasis in Neuron Models. *PLoS Computational Biology*, 10(5):e1003551, 5 2014. ISSN 1553-7358. doi: 10.1371/journal.pcbi.1003551. URL http://dx.plos.org/10.1371/journal.pcbi.1003551.

M. S. Jensen and Y. Yaari. Role of Intrinsic Burst Firing, Potassium Accumulation, and Electrical Coupling in the Elevated Potassium Model of Hippocampal Epilepsy. *Journal of Neurophysiology*, 77 (3):1224–1233, 3 1997. ISSN 0022-3077. doi: 10.1152/jn.1997.77.3.1224. URL http://www.ncbi.nlm.nih.gov/pubmed/9084592http://www.physiology.org/doi/10.1152/jn.1997.77.3.1224.

H. Kager, W. J. Wadman, and G. G. Somjen. Simulated seizures and spreading depression in a neuron model incorporating interstitial space and ion concentrations. *Journal of Neurophysiology*, 84(1), 2000. ISSN 00223077. doi: 10.1152/jn.2000.84.1.495.

H. Kager, W. J. Wadman, and G. G. Somjen. Seizure-like afterdischarges simulated in a model neuron. *Journal of Computational Neuroscience*, 22 (2), 2007. ISSN 09295313. doi: 10.1007/s10827-006-0001-y.

A. E. Moseley, S. P. Lieske, R. K. Wetzel, P. F. James, S. He, D. A. Shelly, R. J. Paul, G. P. Boivin, D. P. Witte, J. Marino Ramirez, K. J. Sweadner, and J. B. Lingrel. The Na,K-ATPase 2 Isoform Is Expressed in Neurons, and Its Absence Disrupts Neuronal Activity in Newborn Mice. 2002. doi: 10.1074/jbc.M211315200. URL `http://www.jbc.org/`.

J. A. Owen, E. Barreto, and J. R. Cressman. Controlling Seizure-Like Events by Perturbing Ion Concentration Dynamics with Periodic Stimulation. *PLoS ONE*, 8(9), 2013. ISSN 19326203. doi: 10.1371/journal.pone.0073820.

L. Øyehaug, I. Østby, C. M. Lloyd, S. W. Omholt, and G. T. Einevoll. Dependence of spontaneous neuronal firing and depolarisation block on astroglial membrane transport mechanisms. *Journal of Computational Neuroscience*, 32(1), 2012. ISSN 09295313. doi: 10.1007/s10827-011-0345-9.

M. J. Sætra, G. T. Einevoll, and G. Halnes. An electrodiffusive, ion conserving Pinsky-Rinzel model with homeostatic mechanisms. *PLoS Computational Biology*, 16(4), 2020. ISSN 15537358. doi: 10.1371/journal.pcbi.1007661.

J.-H. Schleimer, J. Hesse, S. A. Contreras, and S. Schreiber. Firing statistics in the bistable regime of neurons with homoclinic spike generation, 2020.

M. Uzuntarla, J. R. Cressman, M. Ozer, and E. Barreto. Dynamical structure underlying inverse stochastic resonance and its implications. *Physical Review E - Statistical, Nonlinear, and Soft Matter Physics*, 88(4), 2013. ISSN 15393755. doi: 10.1103/PhysRevE.88.042712.

Y. Wei, G. Ullah, J. Ingram, and S. J. Schiff. Oxygen and seizure dynamics: II. Computational modeling. *Journal of Neurophysiology*, 112(2):213–223, 7 2014a. ISSN 0022-3077. doi: 10.1152/jn.00541.2013. URL `http://www.ncbi.nlm.nih.gov/pmc/articles/PMC4064403/`.

Y. Wei, G. Ullah, and S. J. Schiff. Unification of neuronal spikes, seizures, and spreading depression. *Journal of Neuroscience*, 34(35), 2014b. ISSN 15292401. doi: 10.1523/JNEUROSCI.0516-14.2014.
